# Supplementary material for: Formation of β-Strand Oligomers of Antimicrobial Peptide Magainin 2 Contributes to Disruption of Phospholipid Membrane
Source: Membranes (Basel). 2022 Jan 21;12(2):131. doi: 10.3390/membranes12020131 (PMC8877076; doi:10.3390/membranes12020131)
Supplement: Supplementary file 1 [file membranes-12-00131-s001.zip › membranes-1550497-supplementary.pdf]

Supporting Information

Formation of β-Sheet Oligomers of Antimicrobial Peptide Magainin 2 Contributes to Disruption of Phospholipid Membrane

Munehiro Kumashiro[1], Ryoga Tsuji[2], Shoma Suenaga[1], and Koichi Matsuo[1,2,3]

[1]Department of Physical Science, Graduate School of Science, Hiroshima University, 1-3-1 Kagamiyama, Higashi-Hiroshima, Hiroshima 739-8526, Japan

[2]Physics Program, Graduate School of Advanced Science and Engineering, Hiroshima University, 1-4-1 Kagamiyama, Higashi-Hiroshima, Hiroshima, 739-8527, Japan

[3]Hiroshima Synchrotron Radiation Center, Hiroshima University, 2-313 Kagamiyama, Higashi-Hiroshima, Hiroshima 739-0046, Japan

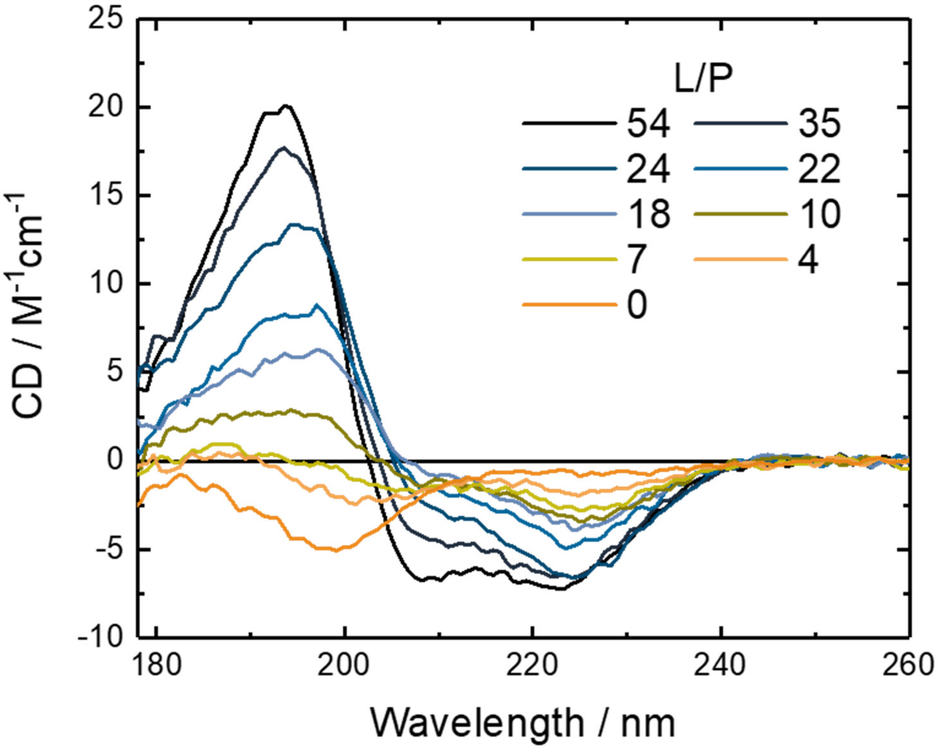

CD / M^{-1}cm^{-1}
Wavelength / nm
L/P
54
35
24
22
18
10
7
4
0
25
20
15
10
5
0
-5
-10
180
200
220
240
260

Figure S1

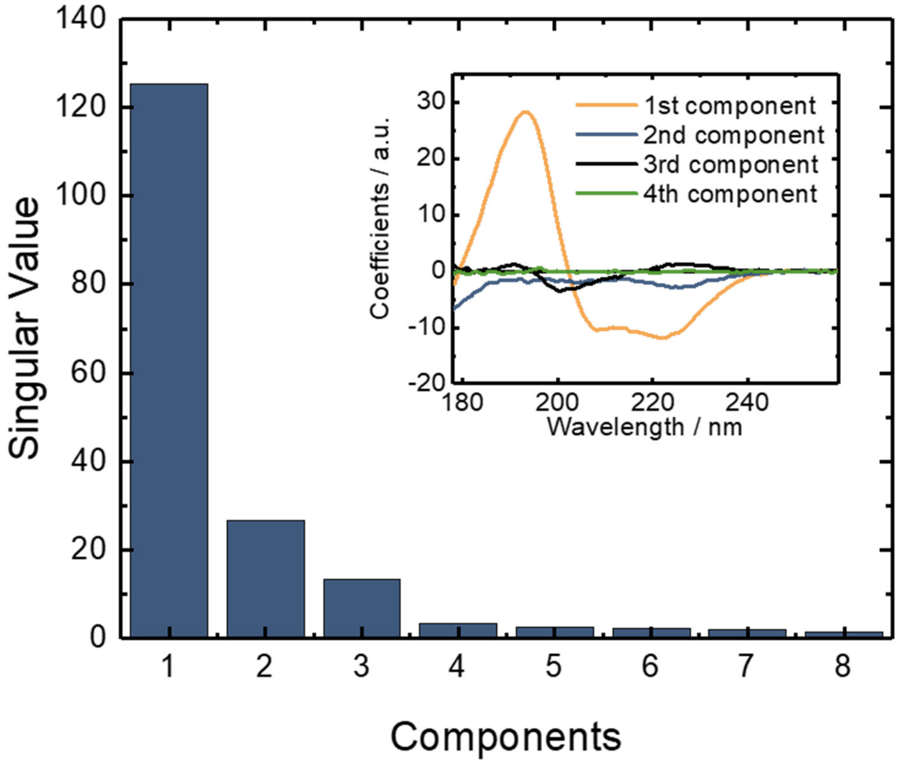
140
120
100
80
60
40
20
0
Singular Value
1
2
3
4
5
6
7
8
Components
30
20
10
0
-10
-20
Coefficients / a.u.
180
200
220
240
Wavelength / nm
1st component
2nd component
3rd component
4th component

Figure S2

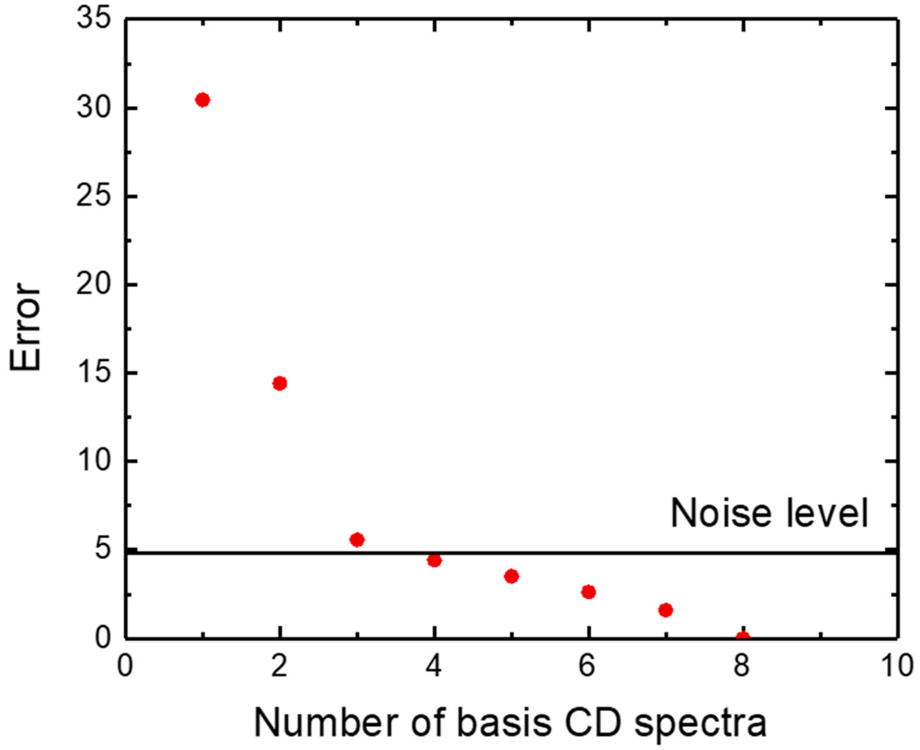
35
30
25
20
15
10
5
0
Error
Noise level
0
2
4
6
8
10
Number of basis CD spectra

Figure S3

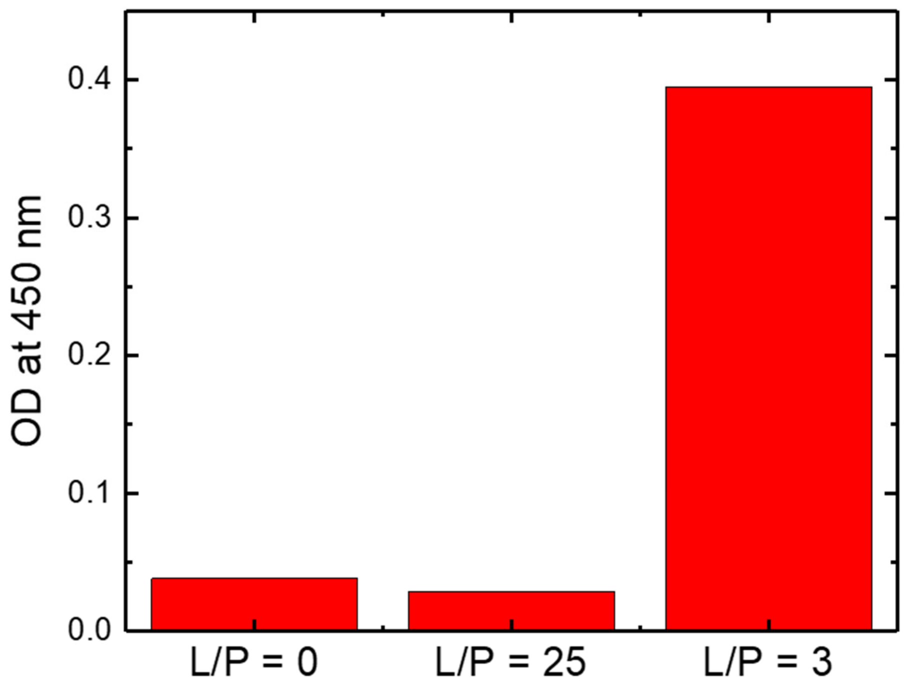

Figure S4

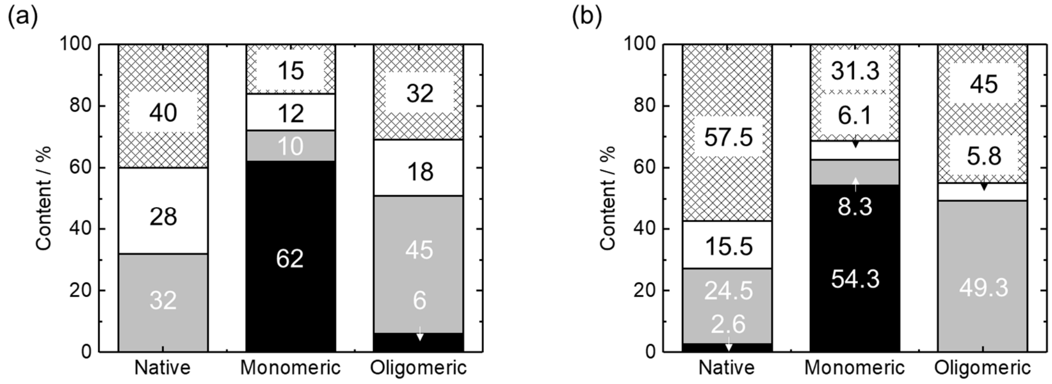
(a)
(b)
Content / %
0
20
40
60
80
100
Native
Monomeric
Oligomeric
40
28
32
15
12
10
62
32
18
45
6
57.5
15.5
24.5
2.6
31.3
6.1
8.3
54.3
45
5.8
49.3

Figure S5

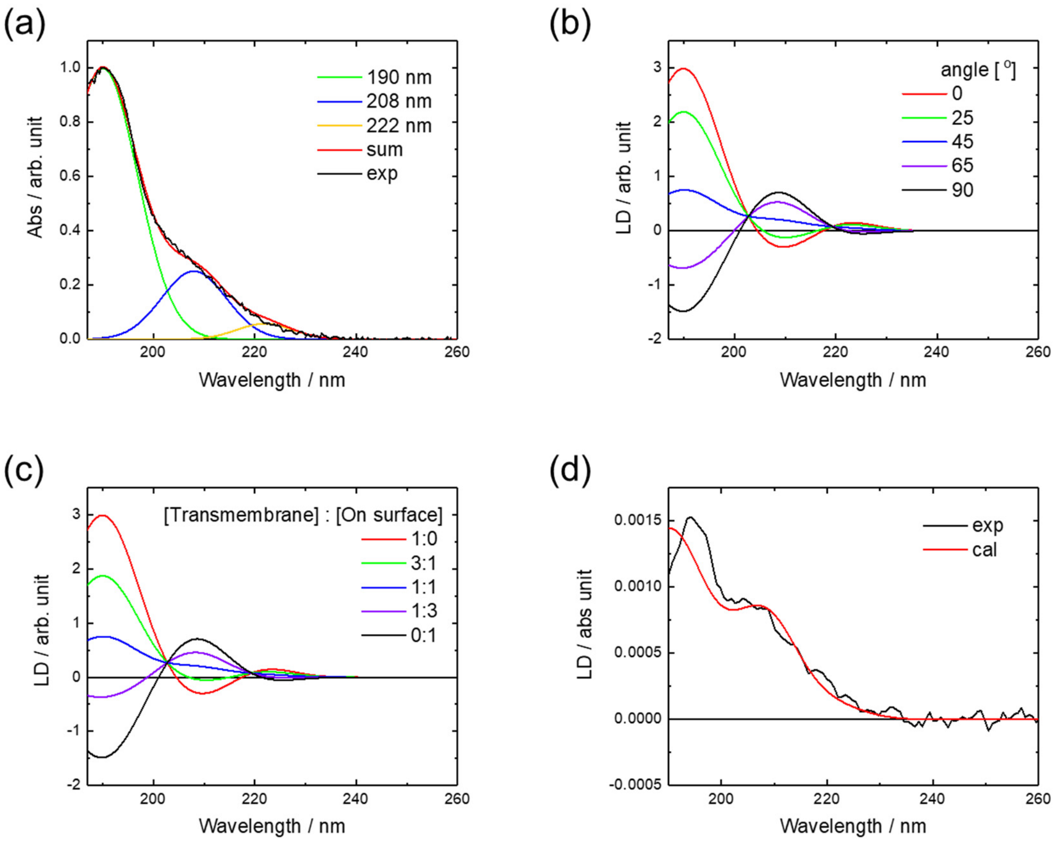
(a)
Abs / arb. unit
Wavelength / nm
190 nm
208 nm
222 nm
sum
exp
(b)
LD / arb. unit
Wavelength / nm
angle [o]
0
25
45
65
90
(c)
LD / arb. unit
Wavelength / nm
[Transmembrane] : [On surface]
1:0
3:1
1:1
1:3
0:1
(d)
LD / abs unit
Wavelength / nm
exp
cal

Figure S6

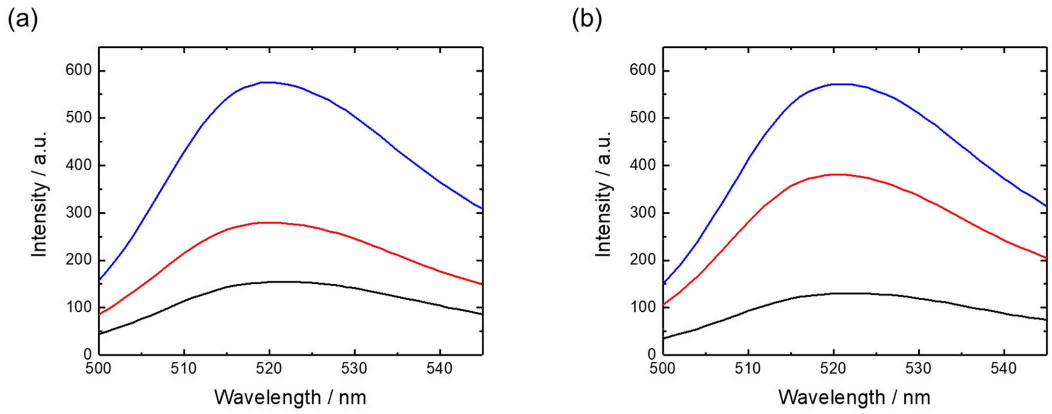

Figure S7

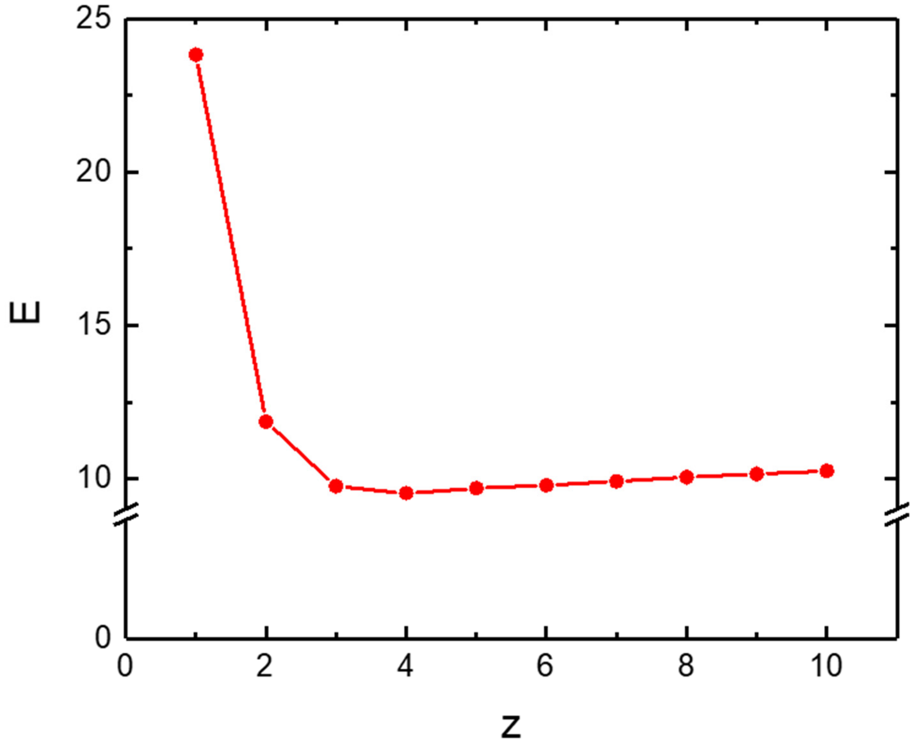

Figure S8

Figure S1: (a) SRCD spectra of M2 in the presence of DPPE/DPPG (molar ratio: 3/1) liposome at the L/P from 0 to 54 at 25 °C

Figure S2: The singular values from the SVD analysis of the SRCD spectra of M2 in DPPG liposomes at 25 °C. In SVD, the data matrix D is decomposed into three matrices as, $D = USV^{\mathrm{T}}$, where U and V are the orthogonal matrices, and S is the diagonal matrix which contains the non-negative singular values on its diagonal. The inset shows the first four rows of US from the SVD analysis. The results showed that only three singular values were significantly larger than zero.

Figure S3: Error in reproducing SRCD data set of M2 in DPPG liposomes using basis CD spectra from SVD analysis. The 2-norm of the error E was calculated by $E = \left|D - U\tilde{S}V^{\mathrm{T}}\right|_2$, where $\tilde{S}$ is the diagonal matrix which contains a part of the singular values obtained from the SVD analysis on its diagonal. Experimental noise level derived from data accumulation was also shown. The noise E_{exp} was calculated by $E_{\mathrm{exp}} = \sqrt{\sum_{k=1}^{l}\sum_{j=1}^{N}\frac{1}{n-1}\sum_{i=1}^{n}\left(\Delta\varepsilon_{ijk} - \overline{\Delta\varepsilon}_{ijk}\right)^2}$, where $\Delta\varepsilon$ is the CD value, $\overline{\Delta\varepsilon}$ is the average CD value, n is the number of data accumulation, N is the number of digitized wavelength points in each spectrum, and l is the number of L/P data points. The results showed that the error calculated using three basis CD spectra was almost equivalent to the experimental noise level.

Figure S4: Optical density at 450 nm of M2-DPPG liposome system at the L/P of 0, 25, and 3 obtained using a commercial absorption spectrophotometer and a quartz cuvette with an optical path length of 1 cm. The concentration of lipid molecule was 400 μM.

Figure S5: Bar graphs of secondary structures of M2 in native, membrane-bound monomeric, and oligomeric states analyzed using (a) CDSSTR and (b) BeStSel programs (black: α-helix; gray: β-strand; white: turn; meshed: unordered structure).

Figure S6: (a) absorption spectrum of M2 in the presence of DPPG liposome at the L/P of 25 obtained by using the VUVCD spectrophotometer and the equation, $\log(HT_{\mathrm{pm}}/HT_{\mathrm{pr}}) - \log(HT_{\mathrm{lm}}/HT_{\mathrm{lr}})$, in which HT_{pm} and HT_{pr} are high tension voltages of the sample of M2 peptides in the presence of DPPG liposomes detected on main photomultiplier (main-PM) and reference photomultiplier (ref-PM), respectively, and

HT_{lm} and HT_{lr} are high tension voltages of DPPG liposome sample detected on main-PM and ref-PM, respectively. The absorption spectrum was deconvoluted into three gaussian-type contributions with peaks at 190 nm, 208 nm, and 222 nm; (b) angle dependence of theoretical LD spectra calculated by using the equation, $A_{LD} = 3/2\, S\Sigma_{n=1}^{3} A_{iso}(\lambda_n)(3\cos^2\gamma(\lambda_n)-1)$ [1], where S is the orientation parameter, A_{iso} is the isotropic absorption spectrum obtained from (a), γ is the angle between the flow and the transition moment directions, and λ_n is the peak wavelength for n-th contribution. For an ideal system, S is equal to 1, meaning that the molecules in the system are completely oriented. The dipoles at 190 nm, 208 nm, and 222 nm of helical peptides are vertical, parallel, and vertical to the helix axis, respectively. The limiting case for a biased alignment behavior is when the elliptical liposome is modeled as an infinite tube, the helical peptides are on the surface of the tube, and the peptides are aligned at an angle α between the helical axis and the normal of the tube surface. In this extreme condition, the γ for LD signals at 190 nm, 208 nm, and 222 nm are 90°, 0°, and 90°, respectively, when the helical peptides are lying parallel to the tube (α=90°). On the other hand, the γ for LD signals at 190 nm, 208 nm, and 222 nm are 0°, 90°, and 0°, respectively, when the helical peptides are inserted perpendicular to the tube (α=0°); (c) [Transmembrane]: [On surface] ratio dependence of the theoretical LD spectra calculated based on the assumption that M2 is in equilibrium between two helix states: transmembrane M2 and M2 on the membrane surface; (d) experimental and calculated LD spectra of M2 in the presence of DPPG liposome at the L/P of 25. The calculated LD spectrum in (d) was obtained by using the optimized angle (= 48°) or [Transmembrane]: [On surface] ratio ([Transmembrane]: [On surface] = 1:1.2).

Figure S7: Fluorescence spectra of calcein in the dye leakage assay before (black) and 40 min after adding M2 solution (red) at L/P = 25 (a) and 4 (b). The blue lines are the fluorescence spectra obtained by adding Triton X-100.

Figure S8: Dependence of the fitting error on the number of subunits z of adsorbed aggregates calculated using fixed K = 17500 and n = 2.2 and K_{1z} as a variable. The minimum point was plotted as a function of z.

1. Rodger, A.; Nordén, B.; Dafforn, T. Linear Dichroism and Circular Dichroism: A Textbook on Polarized-Light Spectroscopy; Royal Society of Chemistry, 2010
